# Supplementary material for: Neutralizing antibodies induced in immunized macaques recognize the CD4-binding site on an occluded-open HIV-1 envelope trimer
Source: Nat Commun. 2022 Feb 8;13:732. doi: 10.1038/s41467-022-28424-3 (PMC8826976; doi:10.1038/s41467-022-28424-3)
Supplement: Supplementary file 1 — Supplementary Information [file 41467_2022_28424_MOESM1_ESM.pdf]

Supplementary Information

Neutralizing antibodies induced in immunized macaques recognize the CD4-binding site on an occluded-open HIV-1 envelope trimer

Zhi Yang[1], Kim-Marie A. Dam[1], Michael D. Bridges[2,3], Magnus A.G. Hoffmann[1], Andrew T. DeLaitsch[1], Harry B. Gristick[1], Amelia Escolano[4], Rajeev Gautam[5], Malcolm A. Martin[5], Michel C. Nussenzweig[4,6], Wayne L. Hubbell[2,3], Pamela J. Bjorkman[1,*]

[1]Division of Biology and Biological Engineering, California Institute of Technology, Pasadena, CA, USA

[2]Jules Stein Eye Institute, University of California, Los Angeles, CA, USA

[3]Department of Chemistry and Biochemistry, University of California, Los Angeles, CA, USA

[4]Laboratory of Molecular Immunology, The Rockefeller University, New York, NY, USA

[5]Laboratory of Molecular Microbiology, National Institute of Allergy and Infectious Diseases, National Institutes of Health, Bethesda, MD, USA

[6]Howard Hughes Medical Institute, Chevy Chase, MD, USA

* Correspondence email: bjorkman@caltech.edu

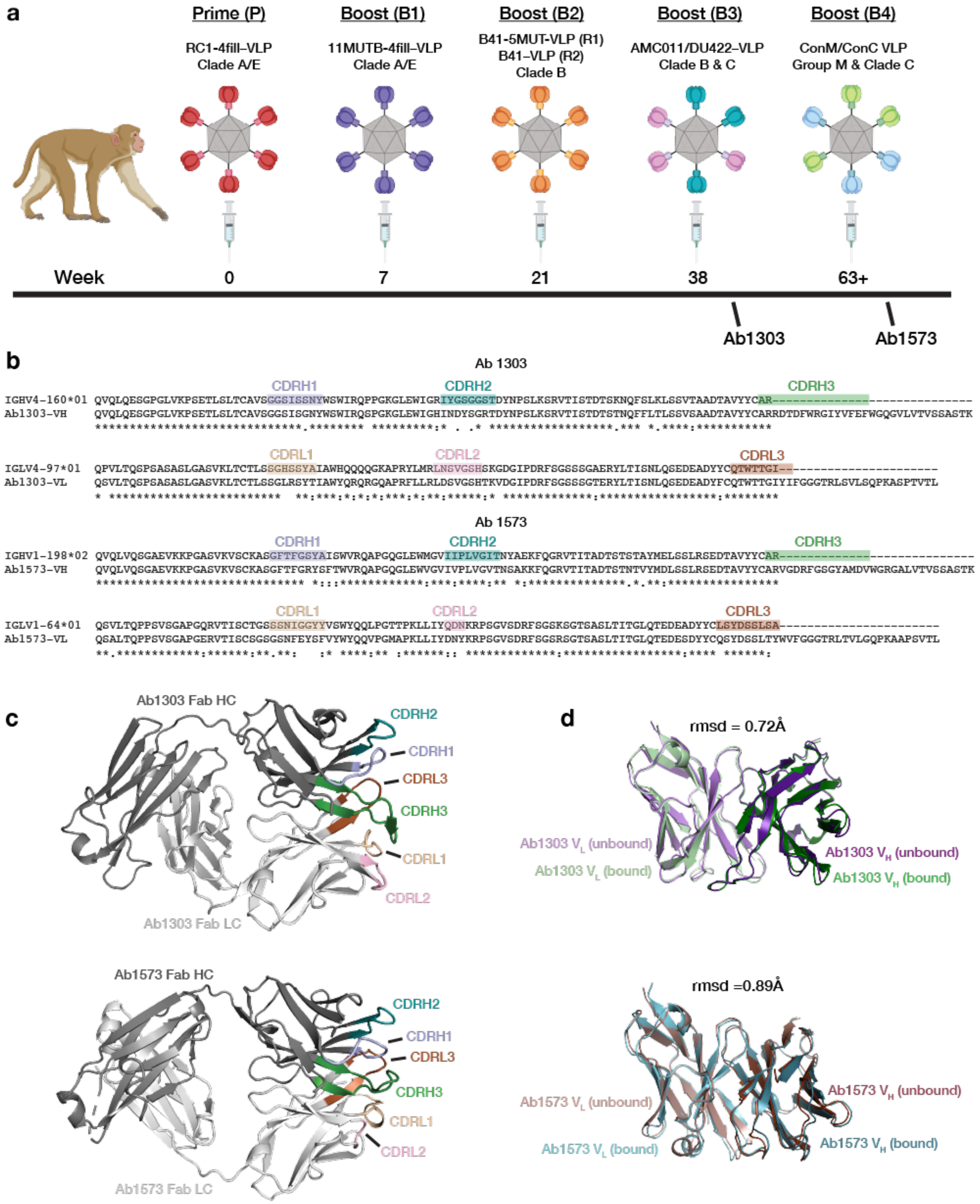

Supplementary Figure 1. Characterization of Ab1303 and Ab1573

(a) Schematic of sequential immunization in NHPs from which Ab1303 (Regimen 1, R1) and Ab1573 (Regimen 2, R2) were derived. Ab1303 was isolated from NHP 4 (T15) after Boost 3;

Ab1573 was isolated from NHP 1 (DGJI) after Boost 4 as described[19]. Representation is created with BioRender.com.
(b) Sequence alignments of Ab1303 and Ab1573 V_H and V_L domains with their germline V gene precursors.
(c) Crystal structures of unliganded Ab1303 and Ab1573 Fabs. CDRs of the two antibodies were highlighted in various colors.
(d) Superimposition of structures of bound (from Fab-Env cryo-EM structures) and free (from Fab crystal structures) V_H-V_L domains.

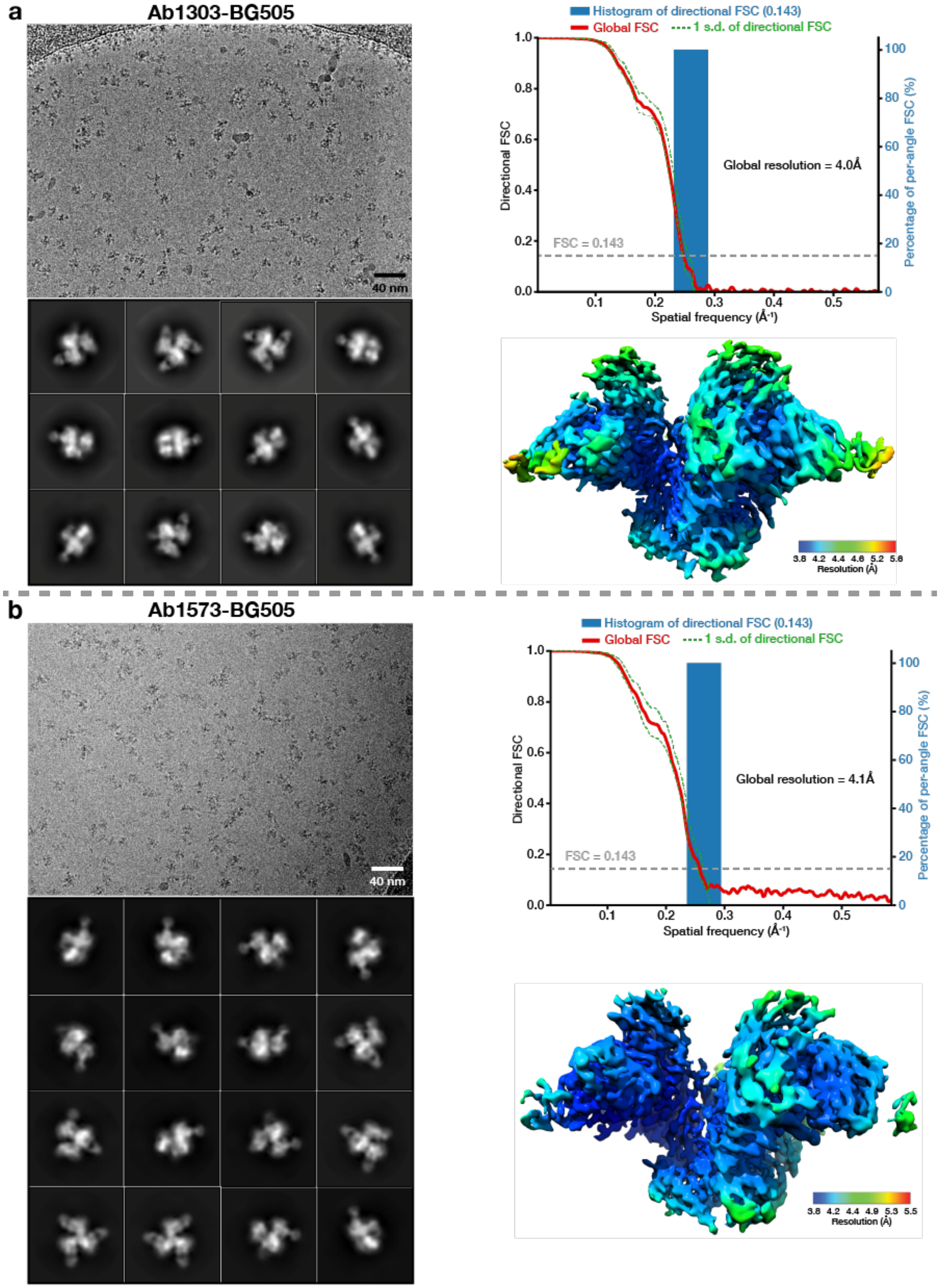

Supplementary Figure 2. Cryo-EM processing, validation and reconstructions
(a,b) Left: Example micrographs and 2D class averages of Ab1303-BG505 (a) and Ab1573-BG505 (b) complexes. Upper right: Plots of global half-map FSCs (solid red line), directional resolution values ±1σ from the mean (left axis, green dashed lines), and histogram distributions sampled over 3D FSC (right axis, blue bars) for Ab1303-BG505 (a) and Ab1573-BG505 (b). Bottom right: local resolution maps of Ab1303-BG505 (a) and Ab1573-BG505 (b).

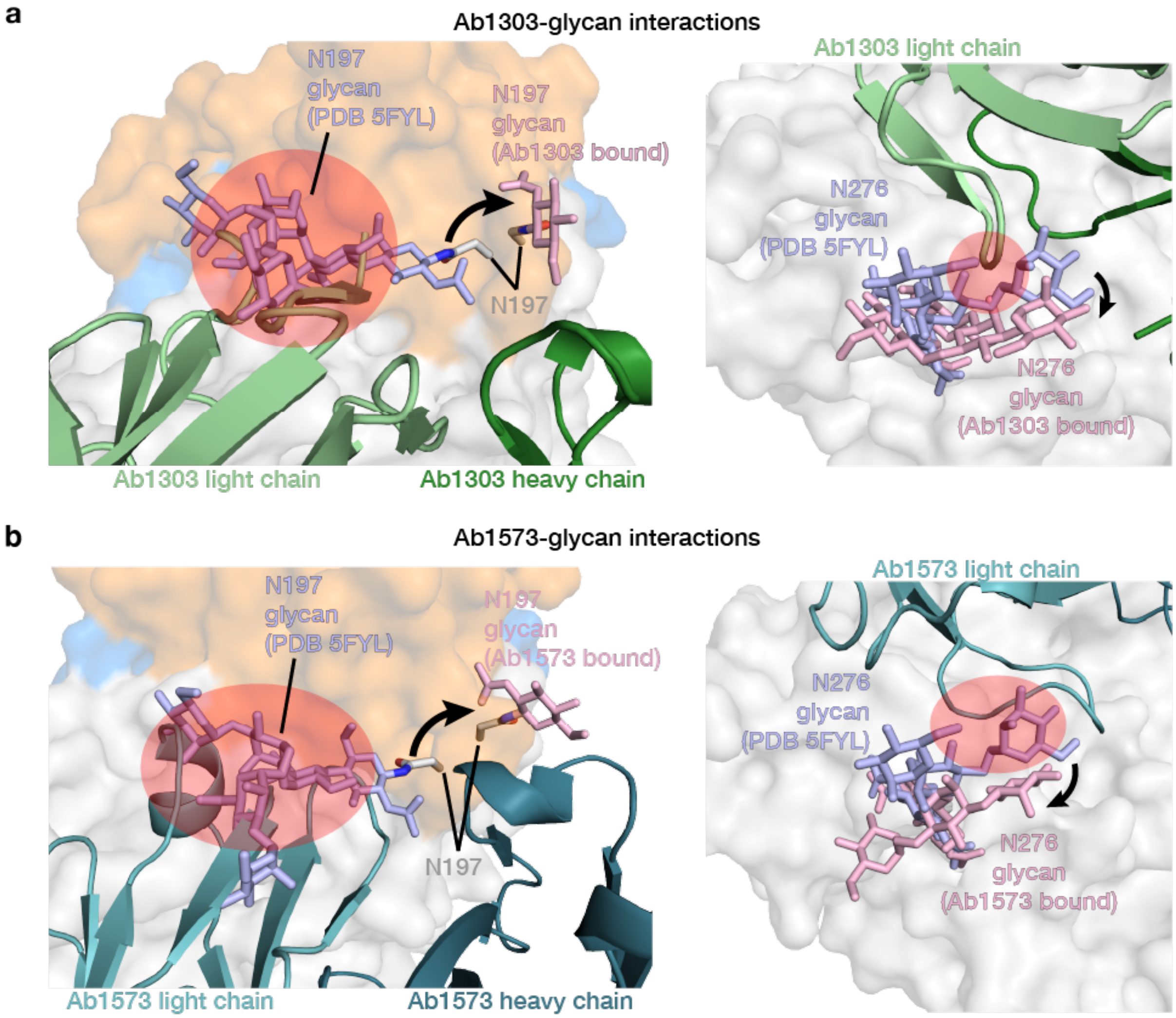

Supplementary Figure 3. gp120 *N*-glycans were displaced to adapt for antibody binding. Locations of $Asn197_{gp120}$ and $Asn276_{gp120}$ glycans (pink) in (a) Ab1303- and (b) Ab1573-bound Env structures were compared with an Env structure that does not have an antibody bound in the vicinity of this region (PDB 5FYL, glycans colored in light blue). Spatially close or clashing regions between the Fabs and glycans in a CD4bs Ab-free trimer are highlighted in red shades. Directions of glycan shifting are indicated with black arrows.

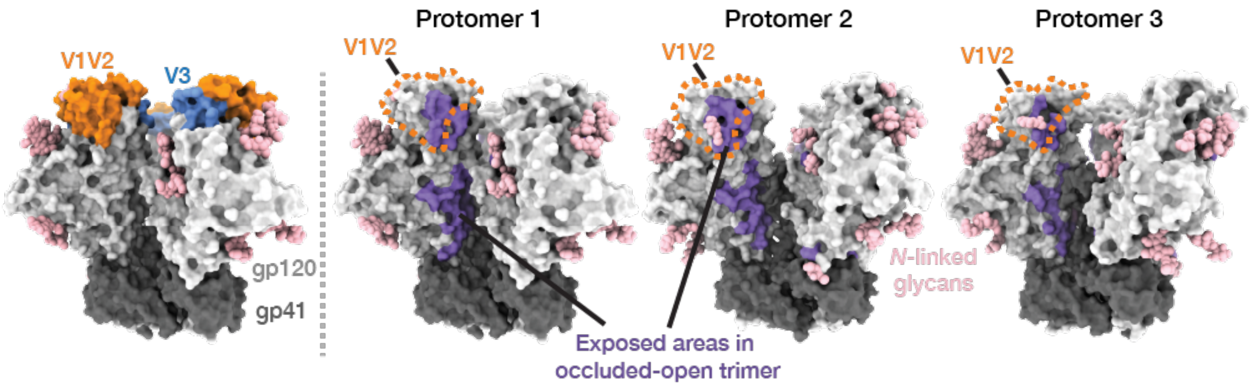

Supplementary Figure 4. gp120 surface area exposed in occluded-open versus closed Env trimers. Left: Occluded-open Env trimer showing V1V2 and V3 regions as colored highlights. Right: Env trimers showing surface areas (purple) that are exposed in occluded-open trimers but buried in closed trimers. Orange dashed shape indicates V1V2 region.

Supplementary table 1. X-ray data collection and refinement statistics (molecular replacement)

	Ab1303 Fab (PDB 7RYU)	Ab1573 Fab (PDB 7RYV)
**Data collection**		
Space group	$P2_12_12_1$	$P2_1$
Cell dimensions		
a, *b*, *c* (Å)	63.85, 66.97, 136.59	81.50 138.40 83.73
α, β, γ (°)	90, 90, 90	90 95.71 90
Resolution (Å)	38.27 - 1.51 (1.53 - 1.51) *	38.91 – 2.50 (2.78-2.50) *
R_{merge} (%)	8.4 (124)	17.4 (98.1)
R_{pim} (%)	2.4 (37.8)	10.8 (62.2)
$CC_{1/2}$ (%)	99.9 (80.2)	99.4 (85.8)
$I / \sigma I$	16.5 (1.2)	8.8 (2.8)
Completeness (%)	99.9 (98.2)	98.3 (95.9)
Multiplicity	13.3 (11.4)	3.6 (3.6)
**Refinement**		
Resolution (Å)	1.51	2.50
No. reflections	92,598 (9,145)	63,132 (6,140)
R_{free} / R_{work} (%)	20.0 / 18.0	25.7 / 22.4
No. atoms		
Protein	3,330	12,931
Ligand/ion	0	0
Water	680	880
R.m.s. deviations		
Bond lengths (Å)	0.008	0.006
Bond angles (°)	0.86	1.15
Rotamer outliers (%)	0	0.34
Ramachandran plot		
Favored (%)	99.3	97.3
Allowed (%)	0.7	2.7
Disallowed	0	0
Average *B*-factor	27.0	30.2

*Values in parentheses are for highest-resolution shell.

Supplementary table 2. Cryo-EM data collection, refinement, and validation statistics

	Ab1303-BG505 (PDB 7TFN) (EMDB-25877)	Ab1573-BG505 (PDB 7TFO) (EMDB-25878)
**Data collection and processing**		
Magnification *	105,000x	105,000x
Voltage (kV)	200	300
Electron exposure (e–/Å^2)	60	60
Defocus range (μm)	1.4-3.0	1.4-3.0
Pixel size (Å)	0.4345	0.4275
Recording mode	Super resolution	Super resolution
Symmetry imposed	C1	C1
Initial particle images (no.)	1,095,744	842,259
Final particle images (no.)	386,825	443,817
Overall map resolution (Å) ** (masked/unmasked)	4.0 (4.4)	4.1 (4.4)
**Refinement**		
Initial model used (PDB code)	5CEZ ***	5CEZ ***
Map and model CC	0.77	0.73
Map sharpening *B* factor (Å^2)	79.3	112
Model composition		
Protein residues	2344	2283
Carbohydrate residues	57	21
Validation		
MolProbity score	2.06	2.23
Clashscore	14.1	16.4
Poor rotamers (%)	0	0
Ramachandran plot		
Favored (%)	93.9	91.1
Allowed (%)	6.1	8.9
Disallowed (%)	0	0
RMS deviations		
Length (Å)	0.003	0.005
Angles (°)	0.63	0.70

* Nominal magnification; ** FSC threshold 0.143; *** Partial structure composed of trimeric gp120/gp41
