# Supplementary material for: Neutralizing antibodies induced in immunized macaques recognize the CD4-binding site on an occluded-open HIV-1 envelope trimer
Source: Nat Commun. 2022 Feb 8;13:732. doi: 10.1038/s41467-022-28424-3 (PMC8826976; doi:10.1038/s41467-022-28424-3)
Supplement: Supplementary file 3 — Description of Additional Supplementary Information [file 41467_2022_28424_MOESM3_ESM.pdf]

Description of Additional Supplementary Information

Title: Supplementary Movie 1

Description: Review of the HIV-1 Env conformational changes among the closed, occluded-open, and CD4- induced fully-open states.”
