# Supplementary material for: Neutralizing antibodies induced in immunized macaques recognize the CD4-binding site on an occluded-open HIV-1 envelope trimer
Source: Nat Commun. 2022 Feb 8;13:732. doi: 10.1038/s41467-022-28424-3 (PMC8826976; doi:10.1038/s41467-022-28424-3)
Supplement: Supplementary file 5 — Reporting Summary [file 41467_2022_28424_MOESM5_ESM.pdf]

nature portfolio

Corresponding author(s): Pamela J. Bjorkman

Last updated by author(s): Jan 12, 2022

Reporting Summary

Nature Portfolio wishes to improve the reproducibility of the work that we publish. This form provides structure for consistency and transparency in reporting. For further information on Nature Portfolio policies, see our Editorial Policies and the Editorial Policy Checklist.

Statistics

For all statistical analyses, confirm that the following items are present in the figure legend, table legend, main text, or Methods section.

n/a	Confirmed	
☐	☒	The exact sample size (*n*) for each experimental group/condition, given as a discrete number and unit of measurement
☐	☒	A statement on whether measurements were taken from distinct samples or whether the same sample was measured repeatedly
☐	☒	The statistical test(s) used AND whether they are one- or two-sided<br>*Only common tests should be described solely by name; describe more complex techniques in the Methods section.*
☒	☐	A description of all covariates tested
☒	☐	A description of any assumptions or corrections, such as tests of normality and adjustment for multiple comparisons
☐	☒	A full description of the statistical parameters including central tendency (e.g. means) or other basic estimates (e.g. regression coefficient) AND variation (e.g. standard deviation) or associated estimates of uncertainty (e.g. confidence intervals)
☒	☐	For null hypothesis testing, the test statistic (e.g. *F*, *t*, *r*) with confidence intervals, effect sizes, degrees of freedom and *P* value noted<br>*Give P values as exact values whenever suitable.*
☒	☐	For Bayesian analysis, information on the choice of priors and Markov chain Monte Carlo settings
☒	☐	For hierarchical and complex designs, identification of the appropriate level for tests and full reporting of outcomes
☒	☐	Estimates of effect sizes (e.g. Cohen's *d*, Pearson's *r*), indicating how they were calculated

Our web collection on statistics for biologists contains articles on many of the points above.

Software and code

Policy information about availability of computer code

Data collection	X-ray data were collected at Stanford Synchrotron Radiation Lightsource (SSRL) beamline 12-1 equipped with an Eiger X 16M detector at a wavelength of 0.9795 Angstrom.<br>Cryo-EM data were collected on a 200kV Talos Arctica (ThermoFisher), or a 300kV Titan Krios electron microscope (ThermoFisher) with a GIF Quantum energy filter (20eV slit), both were equipped with a Gatan K3 direct detector. EM data were collected using SerialEM v3.7 software.<br>Double electron-electron resonance (DEER) spectroscopy data were collected on a Q-band Bruker ELEXSYS 580 spectrometer using a 150 W TWT amplifier (Applied Engineering Systems, Fort Worth, TX) and an E5106400 cavity resonator (Bruker Biospin).
Data analysis	X-ray data were indexed, integrated, scaled in XDS and merged with AIMLESS v0.7.4. The Fab structures determined by molecular replacement using PHASER v2.8.2. Coordinates were refined using Phenix v1.19.2 and iterations of manual building in Coot.<br>Cryo-EM data were processed using RELION v3.1 programs package. Movies were motion-corrected using MotionCor2 with 2x binning, CTFs of motion-corrected micrographs were estimated using CTFFIND v4.1.14, reference-free 2D classifications, 3D classifications, 3D refinements and map sharpening were processed using RELION v3.1. Molecules' interatomic distances were measured in PyMol v2.3 (Schrodinger, LLC).<br>DEER dipolar data were analyzed using LongDistances v.932, a custom program written by Christian Altenbach in LabVIEW (National Instruments); software available online (http://www.biochemistry.ucla.edu/biochem/Faculty/Hubbell/).

For manuscripts utilizing custom algorithms or software that are central to the research but not yet described in published literature, software must be made available to editors and reviewers. We strongly encourage code deposition in a community repository (e.g. GitHub). See the Nature Portfolio guidelines for submitting code & software for further information.

Data

Policy information about availability of data

All manuscripts must include a data availability statement. This statement should provide the following information, where applicable:

- Accession codes, unique identifiers, or web links for publicly available datasets
- A description of any restrictions on data availability
- For clinical datasets or third party data, please ensure that the statement adheres to our policy

The atomic models generated in this study have been deposited in the Protein Data Bank (PDB) under accession codes 7RYU [https://doi.org/10.2210/pdb7RYU/pdb] and 7RYV [https://doi.org/10.2210/pdb7RYV/pdb] for Ab1303 Fab and Ab1573 Fab X-ray crystal structures, respectively. The cryo-EM maps and atomic coordinates for Ab1303-BG505 and Ab1573-BG505 complexes have been deposited in the Electron Microscopy Data Bank (EMDB) with accession codes EMD-25877 and PDB 7TFN [https://doi.org/10.2210/pdb7TFN/pdb], and EMD-25878 and PDB 7TFO [https://doi.org/10.2210/pdb7TFO/pdb], respectively. Source data are provided with this paper.

Field-specific reporting

Please select the one below that is the best fit for your research. If you are not sure, read the appropriate sections before making your selection.

[x] Life sciences [ ] Behavioural & social sciences [ ] Ecological, evolutionary & environmental sciences

For a reference copy of the document with all sections, see nature.com/documents/nr-reporting-summary-flat.pdf

Life sciences study design

All studies must disclose on these points even when the disclosure is negative.

Sample size	Sample sizes and protein concentrations for X-ray crystallography were estimated based on previous literatures and have been shown to be sufficient; sample sizes and protein concentrations for cryo-EM were estimated based on previous experiments and literatures and have been shown to be sufficient; samples sizes for ELISA experiments were estimated for technical duplicates; sample sizes for DEER experiments have been shown to be sufficient based on experience and published literatures.
Data exclusions	Certain outlier data points with background values shown in Figure 1a Left were excluded, the excluded data wouldn't affect the result, and conclusions were further validated from structural perspectives later in the context.<br>Bad particle images were excluded during data processing such as 2D classifications and 3D classifications to generate high-resolution EM maps, the method has been proven to be effective by numerous publications.
Replication	ELISA experiments were performed with n=2 independent biological replicates. Cryo-EM data were recorded and processed multiple times and showed same results with different resolutions. The datasets with the highest resolutions are reported here in this work.
Randomization	Randomization is not relevant to structural data, ELISA, or SEC-MALS.
Blinding	The person who recorded and processed the DEER data was blinded. The person who recorded the ELISA experiments in Figure 1a was blinded. The same group of investigators designed and performed the experiments and analyzed the data for the rest of the results.

Reporting for specific materials, systems and methods

We require information from authors about some types of materials, experimental systems and methods used in many studies. Here, indicate whether each material, system or method listed is relevant to your study. If you are not sure if a list item applies to your research, read the appropriate section before selecting a response.

Materials & experimental systems

n/a	Involved in the study	
[ ]	[x]	Antibodies
[ ]	[x]	Eukaryotic cell lines
[x]	[ ]	Palaeontology and archaeology
[x]	[ ]	Animals and other organisms
[x]	[ ]	Human research participants
[x]	[ ]	Clinical data
[x]	[ ]	Dual use research of concern

Methods

n/a	Involved in the study	
[x]	[ ]	ChIP-seq
[x]	[ ]	Flow cytometry
[x]	[ ]	MRI-based neuroimaging

Antibodies

Antibodies used	Antibodies used include anti-HIV Env Fabs Ab1303 (this study), Ab1573 (this study), IOMA, b12, 10-1074, VRC34, PG16, 3BNC117 and

IgGs Ab1303, Ab1573, PGT145; HRP-conjugated anti-human Fc antibodies with goat (Southern Biotech) and mouse (GenScript) origins were also used. Details of the usage are described in the Method section of this paper.

Validation

Binding affinities for anti-HIV Env antibodies (other than the two studied in this paper) have been shown in previous literatures[1-4], binding mechanisms have been characterized through structural studies[5-6]. Binding specificities of anti-human Fc antibodies have been validated by commercial source (information can be found at: Southern Biotech https://www.southernbiotech.com/?catno=2048-05&type=Polyclonal#&panel2-1; and GeneScript A00186: https://www.genscript.com/antibody/A00186-THE_sup_TM_sup_His_Tag_Antibody_mAb_Mouse.html), effectiveness of these commercial antibodies also have been repeatedly tested in ELISA experiments in our lab.

References:
1. Scheid, J.F. et al. Sequence and Structural Convergence of Broad and Potent HIV Antibodies That Mimic CD4 Binding. Science 333, 1633-1637 (2011).
2. Mouquet, H. et al. Complex-type N-glycan recognition by potent broadly neutralizing HIV antibodies. Proc Natl Acad Sci U S A 109, E3268-77 (2012).
3. Walker, L.M. et al. Broad and Potent Neutralizing Antibodies from an African Donor Reveal a New HIV-1 Vaccine Target. Science 326, 285-289 (2009).
4. Kong, R. et al. Fusion peptide of HIV-1 as a site of vulnerability to neutralizing antibody. Science 352, 828-33 (2016).
5. Gristick, H.B. et al. Natively glycosylated HIV-1 Env structure reveals new mode for antibody recognition of the CD4-binding site. Nat Struct Mol Biol 23, 906-915 (2016).
6. Ozorowski, G. et al. Open and closed structures reveal allostery and pliability in the HIV-1 envelope spike. Nature 547, 360-363 (2017).

Eukaryotic cell lines

Policy information about cell lines

Cell line source(s)	Expi293F cells (ThermoFisher)
Authentication	Cell line was not authenticated in the lab.
Mycoplasma contamination	No contamination.
Commonly misidentified lines (See ICLAC register)	No commonly misidentified lines were used.
